# Identifying Transformative Sequences in the Psychotherapeutic Interaction With Chinese Adolescents With Depression: A Conversation Analysis Approach

**DOI:** 10.3389/fpsyg.2022.810371

**Published:** 2022-06-20

**Authors:** Wen Ma, Xingang Fan, Shuai Zhang

**Affiliations:** ^1^School of Foreign Languages and Literature, Shandong University, Jinan, China; ^2^Language Sciences Lab, Shandong University, Jinan, China; ^3^Department of Psychiatry, The Fourth People’s Hospital, Liaocheng, China; ^4^School of Foreign Languages, Yantai University, Yantai, China

**Keywords:** psychotherapy, conversation analysis, adolescent, depression, transformation

## Abstract

Previous studies seldom touch on aspects of psychotherapeutic encounters between therapists and clients with particular disorders (such as depression). Little attention has been paid to the sequence organization of psychotherapeutic interaction between therapists and clients with depression in Chinese medical settings. By adopting conversation analysis, we investigated the specifics of psychotherapeutic encounters, specifically, the transformative sequences of psychotherapeutic interaction between therapists and Chinese adolescents with depression. We identified the fourth aspect of clients’ experience transformed in the Chinese psychotherapeutic interaction with adolescents with depression: cognition, and described how the sequential organization of therapists’ actions facilitates the momentary transformation of clients’ experience (i.e., the transformation of cognition, referent, emotion, and relation) in the psychotherapeutic processes. This study not only adds to the conversation analytic study on the transformative sequences in psychotherapeutic interaction but also sheds some light on the study of how therapists transform clients’ experience in Chinese psychotherapeutic interaction.

## Introduction

Depression is one of the leading causes of disability worldwide (Ribeiro et al., 2018) and the primary reason for suicidal behavior (Simon, 2003). Depression has become one of the most common mental disorders in adolescents worldwide, with an increasing prevalence (Zhou et al., 2020; Daly, 2022). Depression has exerted an adverse influence on adolescents’ behaviors, such as poor academic performance, suicidal ideation, and substance abuse (e.g., Keenan-Miller et al., 2007; Luk et al., 2010; Owens et al., 2012; Verboom et al., 2014; Earnshaw et al., 2017), and has negative consequences for adolescents’ physical, psychological, and social development (Merikangas et al., 2010; Clayborne et al., 2019). In the United States, the prevalence of depression in adolescents approximately doubled in the past decade (from 8.1% in 2009 to 15.8% in 2019) (Daly, 2022). In China, children and adolescents have suffered relatively different levels of depression at different ages, with an increased prevalence of depression among adolescents in senior middle schools (from 24.5% in Grade 1 to 40.1% in Grade 3) (Tang et al., 2020). In China, researchers have been investigating the influence factors and management of depression in Chinese adolescents (e.g., Lian et al., 2016; Liu et al., 2017; Chen et al., 2018; Cheng and Zhang, 2018; Miao et al., 2018; Sun et al., 2019).

Psychotherapy is considered the management and treatment of psychological disorders (such as depression) through talk (Peräkylä et al., 2008). In psychotherapeutic encounters, therapists, and clients collaboratively achieve psychotherapeutic goals, such as treating client’s psychological disorders and helping clients overcome their behavioral, socio-emotional, and relational obstacles, in the psychotherapeutic process (Peräkylä, 2019; see also Bercelli et al., 2008; Voutilainen et al., 2011). Psychotherapy is characterized by treating disorders of mind and changes in clients’ emotions, behaviors, and social relations (Antaki, 2008; Peräkylä et al., 2008; Peräkylä, 2019). Conversation analysis (CA) is a qualitative approach to examine the orderliness and sequences of naturally occurring interpersonal interactions and analyze how participants collaboratively accomplish social actions through interactional practices (Stivers and Sidnell, 2012). CA is a unique and valuable method to examine the therapeutic changes in clients’ feelings and emotions in psychotherapeutic encounters, with the focuses not only on the sequence organization of psychotherapy but also on the therapeutic changes in clients’ experience (e.g., referents, emotions, and relations) moment by moment in and through sequentially organized actions (such as questions, formulations, and interpretations) in the psychotherapeutic processes (Peräkylä et al., 2008; Voutilainen et al., 2011; Bercelli et al., 2013; Peräkylä, 2019). To date, however, there remains a paucity of work that has applied CA to examine the specifics of psychotherapeutic interactions *in situ* between therapists and clients with particular disorders, except a few studies, such as Muntigl’s (2016) work on the aspects of therapeutic interactions with depressed clients and Mikesell and Bromley’s (2016) work on the aspects of the talk of clients with schizophrenia (Peräkylä, 2019). By adopting CA, our study aims to extend the understandings of the transformative sequences, in and through which clients’ experiences are transformed, in the psychotherapeutic interaction with Chinese adolescents with depression.

## Background

Conversation analysis provides a unique method for investigating the features of psychotherapeutic interaction between therapists and clients (Peräkylä et al., 2008; Peräkylä, 2019). Since the pioneering work of Davis (1986) on problem formulations in therapist–client interaction, there has been a growing body of CA research on the sequentially organized actions and therapeutic changes in clients’ experience in psychotherapeutic interaction (e.g., Peräkylä et al., 2008; Voutilainen et al., 2011; Bercelli et al., 2013; Peräkylä, 2019). The CA studies on the sequence organization and interactional structures of psychotherapeutic interaction are firmly based on the previous CA research on the generic structures (Schegloff, 2007) of social interaction (Antaki, 2008; Peräkylä et al., 2008; Peräkylä, 2019).

The most fundamental unit of sequence organization is the adjacency pair (including the first pair part and the second pair part) (Schegloff, 2007). The nature of the adjacency (or the nextness) plays a central role in the organization and understandings of social interaction (Schegloff, 2007). Prospectively, the first pair part projects and constrains the next turn, while retrospectively, the second pair part provides type-specific responses and shows the speaker’s understandings of the prior turn (Heritage and Atkison, 1984; Schegloff, 2007). The default adjacency pair (i.e., the second pair part after the first pair part) can be expanded into pre-, insert, and post-expansions (Schegloff, 2007). For example, as one of the sequential patterns of conversational interaction, the following three-turn (action) structure has been widely investigated in CA research on psychotherapeutic interaction (see also Peräkylä, 2019):

(1) First pair part (Initiation).
(2) Second pair part (Response).
(3) Third-position action.

Many CA studies have been conducted on the actions of this three-turn (action) structure. For example, by investigating the online cognitive behavioral therapeutic interaction between therapists and clients, Ekberg et al. (2013, 2016) examined the question-answer-third-position actions (i.e., the actions follow the clients’ responses to therapists’ questions) and found three types of third-position actions: *thanking*, *commiseration* and *emotional inference*. Clients’ answers informed and made these three types of responses relevant in the second position, displaying the sequential focus of the psychotherapeutic interaction (Ekberg et al., 2013, 2016; Peräkylä, 2019).

The sequential structures of the psychotherapeutic interaction enable and facilitate the transformation of clients’ experience (i.e., the transformation of referent, emotion, and relation) in and through sequentially organized social action, such as formulations, interpretations, and questions (Peräkylä, 2019; see also Peräkylä et al., 2008; Voutilainen et al., 2011; Bercelli et al., 2013). One of the most-researched third-position actions in CA studies on psychotherapeutic interaction is the formulation (Peräkylä, 2019; see also, e.g., Antaki, 2008; see also Vehvilainen, 2003; Antaki et al., 2005; Hutchby, 2005; Bercelli et al., 2008; Weiste and Peräkylä, 2013). By formulating, participants propose their own (candidate) understandings of the other speaker’s prior talk with some changes (Garfinkel and Sacks, 1970; Heritage and Watson, 1980). Formulations make relevant the responses like (dis)confirmation, (dis)agreement, and elaboration by the other speaker (Schegloff, 2007; Antaki, 2008; Weiste and Peräkylä, 2013; Peräkylä, 2019). In psychotherapeutic interaction, therapists and clients can formulate particular aspects of the other’s subjective experiences and emotional descriptions. Weiste and Peräkylä (2013) proposed four types of therapists’ formulations of clients’ reports of their experiences: highlighting formulations, rephrasing formulations, relocating formulations, and exaggerating formulations, with different tasks, linguistic features, and sequential features.

The sequential patterns of psychotherapeutic interaction not only show the interactional details of therapist–client interaction (i.e., how social actions are collaboratively accomplished, how therapists’ actions constrain clients’ responses, and how therapists respond to clients’ resistance or disagreement), but also demonstrate the socio-psychological changes in clients (such as feelings, emotions, and relations) in the psychotherapeutic process (Peräkylä et al., 2008; Voutilainen et al., 2011; Bercelli et al., 2013; Peräkylä, 2019). Peräkylä (2019) demonstrates that the transformation of experience is accomplished through the sequentially organized social actions in the psychotherapeutic interaction and provides a detailed explanation for the model and the sequential relations. In the model, *Target action* means the focus of the analyses of the social actions transforming clients’ experience, such as formulations, questions, repair, and interpretations. *Prior action* refers to the actions before target action, which affords and constrains the target action. *Response* is made relevant by the target action and provided by the other participant (i.e., the client in our study). *Third position* refers to the actions performed by the producer (i.e., the therapist in this study) of the target action in response to the other participant’s (i.e., the client in our study) response. In this process, the transformation of clients’ experience (including referents, emotions, and relations) is incorporated in the sequence of adjacent utterances and displayed in and through sequentially organized actions (Voutilainen et al., 2011; Bercelli et al., 2013; Peräkylä, 2019).

The transformative sequences are recognized as a vehicle for foregrounding and transforming clients’ experience in psychotherapy, thus helping clients to understand and overcome their emotional, relational, cognitive and psychological difficulties and facilitating the momentary changes in clients’ emotions, psychological, and social behaviors (Peräkylä et al., 2008; Voutilainen et al., 2011; Bercelli et al., 2013; Peräkylä, 2019). However, to our knowledge, there is no CA study on the transformative sequences in Chinese psychotherapeutic interaction and on the features of the Chinese psychotherapeutic interaction between therapists and clients with particular disorders (such as depression). Depression is a common and severe mental disorder among adolescents globally and has become a major health concern for adolescents globally (e.g., Eapen and Crncec, 2012; Daly, 2022). CA studies are needed to explore the features and structures of the moment-by-moment interaction and the therapeutic management of the clients’ depression in the psychotherapeutic interaction between Chinese therapists and adolescents with depression. This study investigates the transformative sequences of the psychotherapeutic interaction between therapists and Chinese adolescents with depression. Specifically, this study aims to address the following questions: (1) What are the features of the transformative sequences in the psychotherapeutic interaction between therapists and adolescents with depression? (2) How clients’ experience is transformed through sequentially organized actions? Our study contributes to the CA studies on the organization of transformative sequences and the transformation of experience in Chinese psychotherapeutic interaction.

## Materials and Methods

Our research group collected the data from March 2017 to April 2021 for a larger project to examine therapist–client interaction in the Chinese psychological counseling clinic in a tertiary hospital in Shandong Province in northeast China. The dataset included 9 full-time therapists and 136 clients from the same province. Among these interactions, we recorded 15 encounters between three therapists (two males/one female) and seven Chinese adolescents with depression (three males/four females; aged 14–17 years) in the outpatient clinic. The demographic information about Chinese adolescents with depression is presented in Table 1. Each encounter lasted approximately 50–85 min, averaging 63 min. We obtained written consent from all participants before collecting the data. We used pseudonyms for persons and places in the data to ensure anonymity.

**TABLE 1:** Demographic information about Chinese adolescents with depression.

Clients Demography	Chinese adolescents with depression						
	C1	C2	C3	C4	C5	C6	C7
							
Gender	Female	Male	Female	Female	Male	Female	Male
Age	15	17	14	16	15	16	16
Number of visits	2	2	2	2	3	2	2
Total duration (min)	100	136	151	144	141	170	106
Therapists	T1	T1	T2	T1	T3	T3	T2
Location	Shandong Province	Shandong Province	Shandong Province	Shandong Province	Shandong Province	Shandong Province	Shandong Province

The analytical method for this study is CA (Stivers and Sidnell, 2012), since it has become a practical and unique approach for examining the sequence organization of therapist–client encounters and describing momentary changes in feelings, emotions, and relations in naturally occurring psychotherapeutic interaction (Peräkylä, 2019). CA is a qualitative approach to examine the orderliness and sequences of naturally occurring recorded interpersonal interactions and analyze how participants achieve shared intersubjective understandings and accomplish social actions through interactional practices (Schegloff, 2007; Stivers and Sidnell, 2012). It investigates how participants design their talks to accomplish specific actions and yields data-based and empirical-grounded claims about the features of talk-in-interaction (Stivers and Sidnell, 2012). CA studies involve at least four steps: audio-/video-recording and transcribing social interactions, analyzing the data, and reporting findings (ten Have, 2007). Taking an emic perspective, conversation analysts conduct inductive analyses of the recordings in an unmotivated way to find out recurrent patterns and underlying rules and principles of interaction (Sacks et al., 1974).

After collecting the data, all three authors transcribed the data based on Jefferson (2004) transcription convention (see Appendix A) and the Leipzig Glossing Rules.^1^ We presented the extracts with a four-line system: (1) Chinese verbatim, (2) pinyin orthography, (3) word-by-word glosses, and (4) idiomatic English translation. In the extracts, C is the abbreviation of *client* and T is *therapist*. We then sorted, identified, and summarized the regularities and patterns in the data in an inductive way. After careful observation and detailed examination of the recordings and the transcribed data, we observed the therapists’ transformative sequences in the data. Through detailed sequential analysis, we identified three recurrent types of the transformative sequences that transform four aspects of clients’ experience in Chinese psychotherapeutic interaction between therapists and adolescents with depression.

## Analysis

By adopting CA as the analytical approach, we investigated the features of the transformative sequences of the psychotherapeutic interaction between Chinese therapists and adolescents with depression. By taking an emic perspective and scrutinizing the data, unlike the work of Peräkylä (2019), we identified the fourth aspect of clients’ experience transformed in the Chinese psychotherapeutic interaction: cognition. Cognition involves the changes in the clients’ perceptions and understandings of their emotional, psychological, and cognitive difficulties and problems. We further demonstrated that the momentary changes in cognition, referents, emotions, and relations are involved and accomplished in and through the transformative sequences, thus helping clients face and overcome their emotional, cognitive, and psychological difficulties and problems.

The sequences of actions document and transform the momentary display of referents, emotion, relation, and cognition in the psychotherapeutic process. This is the psychotherapeutic project: the sequence organization of the psychotherapeutic interaction in which actions are collaboratively developed and performed to help clients face and overcome their psychological difficulties and problems (Peräkylä, 2019). In the following sections, we demonstrate how these transformative sequences enable the transformation of experience and facilitate the changes in clients’ cognition, referents, emotions, and relations.

### Transformation of Cognition

The transformation of cognition involves the changes in the clients’ perceptions and understandings of their emotional, psychological, and cognitive difficulties and problems. In the psychotherapeutic interaction, the transformative sequences enable therapists to promote the changes in clients’ cognitive positions, particularly from being unknowing and unaware to being knowing and aware of their emotional and psychological problems. The following Extract 1 demonstrates how the therapist manages and transforms the client’s cognitive position.

**Extract 1 (G21429: perfection)**


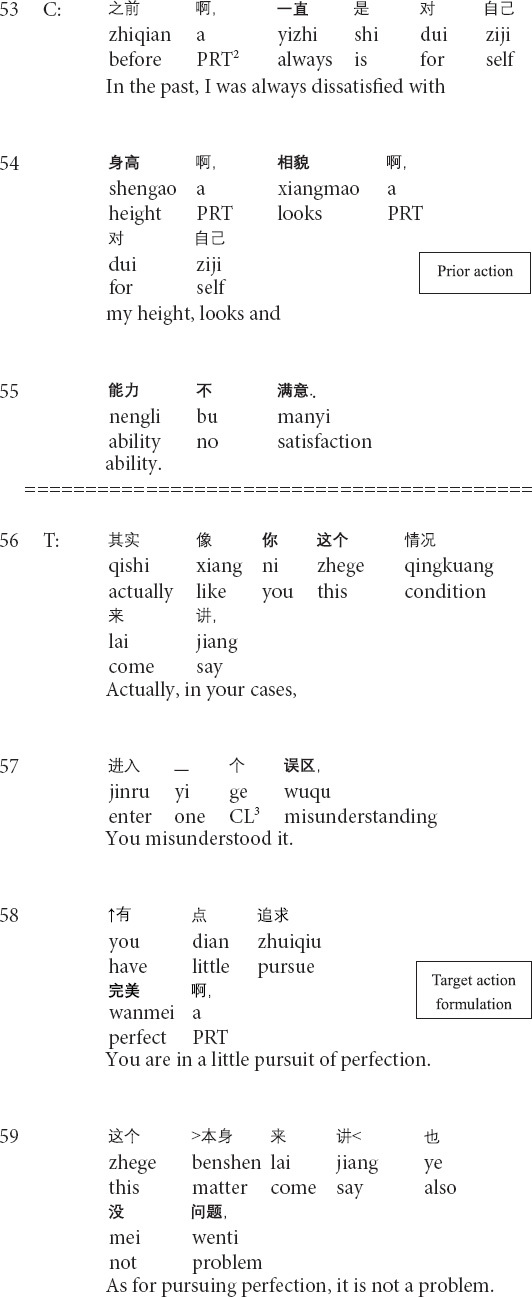



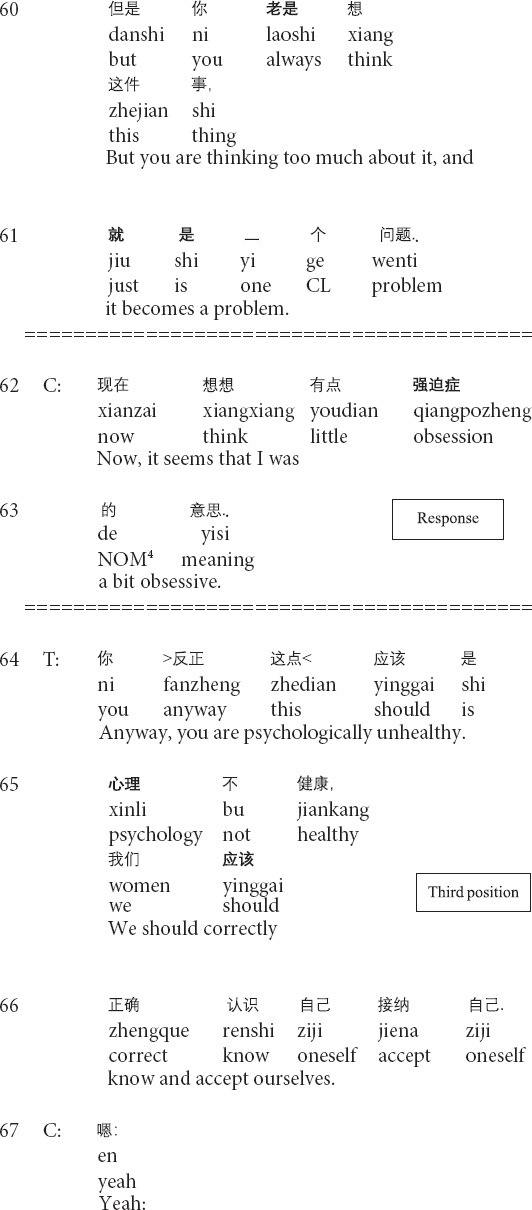


The prior action involves the client’s description of the dissatisfaction with herself (lines 53–55). In lines 56–58, the therapist produces a formulation: focusing on the client’s description of her negative subjective assessment of herself and providing his interpretation (see *relocating formulation* in Weiste and Peräkylä, 2013). Then the client topicalizes the unconscious meanings of the client’s description (Weiste and Peräkylä, 2013) and explicitly presents the client’s psychological behaviors as problematic (lines 59–61). In response, the client not only acknowledges the existence of the psychological problem but also tentatively provides her understandings of the type of the problem by using psychological terms (lines 62–63). At this moment, the therapist’s formulation and interpretation enable the change in the client’s cognitive position: from being unaware to aware of the psychological problem. In the third position, the therapist explicitly points out the client’s unhealthy psychology and proffers the correct way for changing the current unhealthy conditions (64–66). The therapist’s transformation of cognition is therapeutically significant for the client to know, understand and face her psychological problems, thus paving the way for later management and treatment.

### Transformation of Referents

In psychotherapeutic interaction, therapists and clients normatively use various referential terms to refer to different aspects of clients’ experiences in the natural and imagined worlds in and out of the present interactions (Peräkylä, 2019). Therapists adopt several methods in systematic and organized ways, such as questions, interpretations, repair, and formulations, to discuss and transform referents (see, e.g., Peräkylä, 2011, 2019). The following Extract 2 demonstrates how the therapist alters the referents in and through the interpretive formulation, which means that the therapist delivers his points of view about the meanings of the client’s talk (Bercelli et al., 2008; Weiste and Peräkylä, 2013). Before this extract, the client describes his recent behaviors and experiences, which are presented as the reason for the visit.

**Extract 2 (G17314: precocity)**


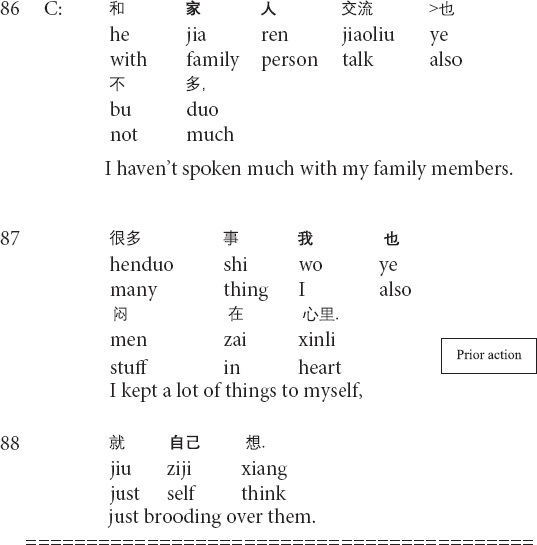



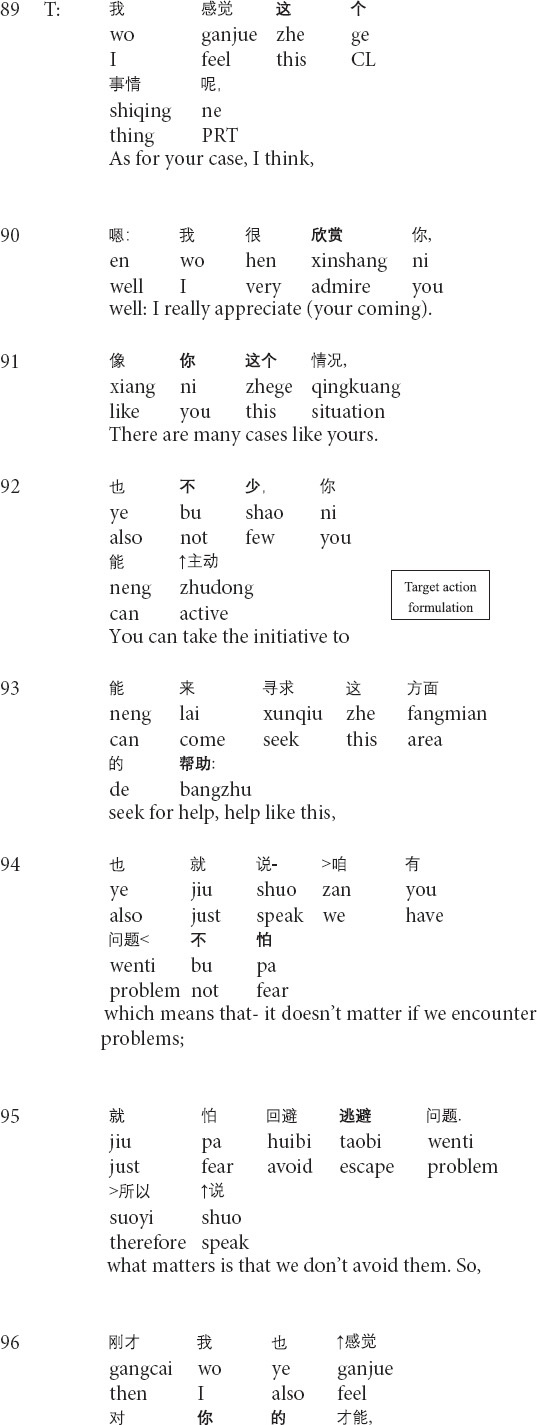



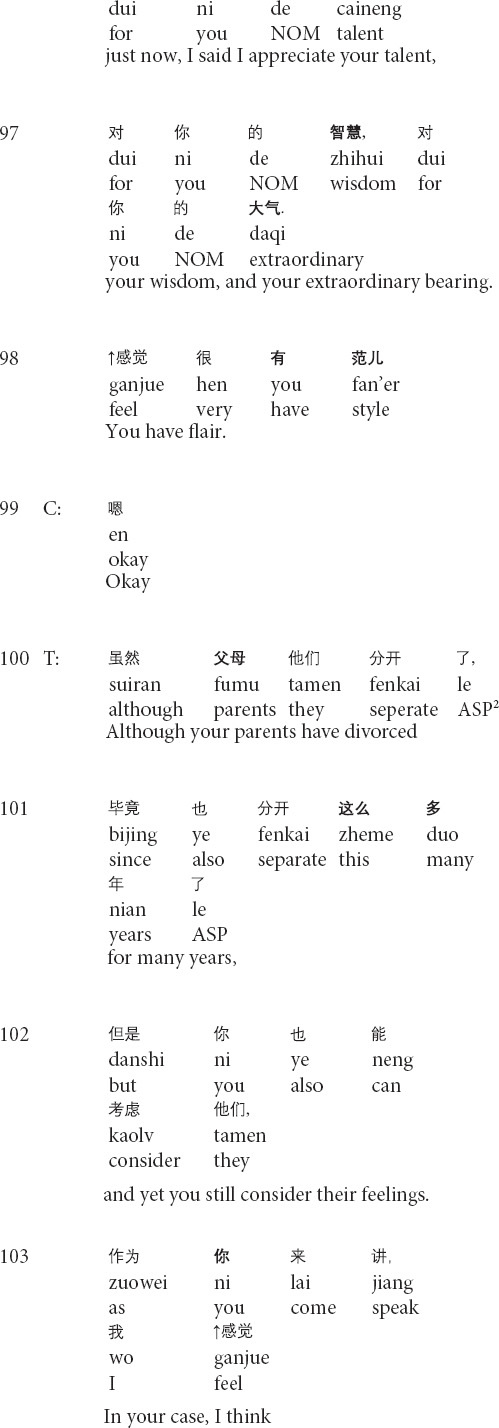



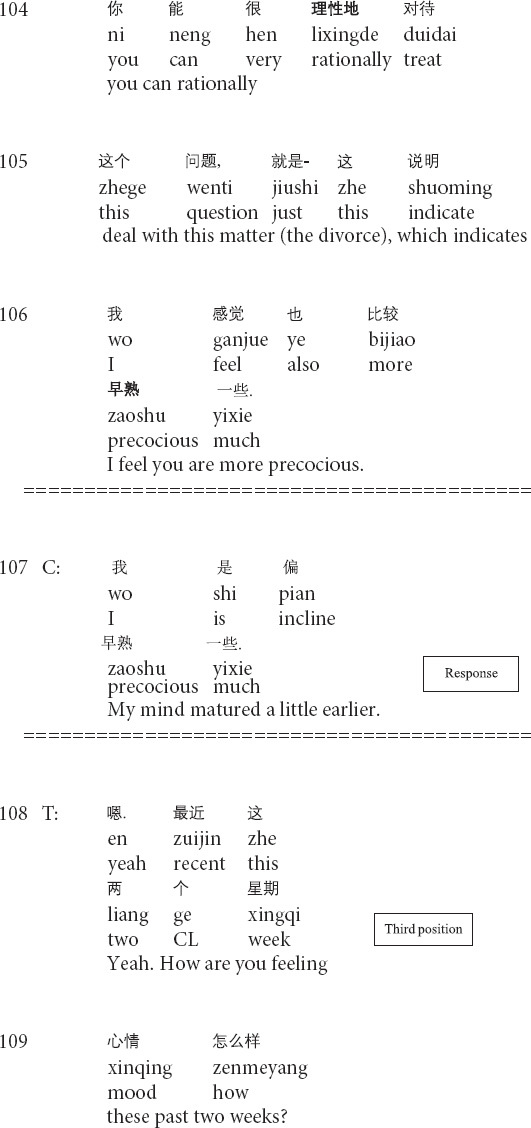


In the beginning part of his responses to the client’s narratives, the therapist prepares his interpretation by introducing the part of the topic in line 89, projecting the general interpretation for the client’s current condition (Vehvilainen, 2003; see also *relocating formulation* in Weiste and Peräkylä, 2013). However, with the turn-initial *well*, the therapist changes the referents from the client’s case to the client’s behaviors and particular aspects of his personality by shifting the focus on talking about the client’s case to complimenting the client (lines 90–98), as a way to reinforce the client’s behaviors of seeking therapeutic help (Jager et al., 2015). After receiving the client’s response, the therapist produces another formulation and changes the referents to topicalize the client’s parent’s divorce (lines 100–101). Then the therapist shifts the focus back to his views and positive evaluations of the client’s behaviors (lines 102–105). By invoking the client’s behaviors and the positive assessment, the therapist grounds his evaluations of the client’s psychological states by adopting the technical term *precocity*. In response, the client displays the agreement with the therapist’s judgment of precocity yet provides his judgment by downgrading from *more* in line 106 to *a little* in line 107. In his third-position action (lines 108–109), the therapist first agrees with the client’s formulation and then starts to inquire about the client’s mood for the past two weeks. Therefore, the transformation of referents topicalizes different aspects of the client’s experiences, thus steering the client to face his psychological problems while gradually realizing his own merits, and reinforcing the client’s desired behaviors (Jager et al., 2015).

Similarly, the following Extract 3 also demonstrates the transformation of referents across the talk. Before Extract 3, the therapist inquires about the post written by the client and the client’s suicidal thoughts.

**Extract 3 (F21425: goodbye)**


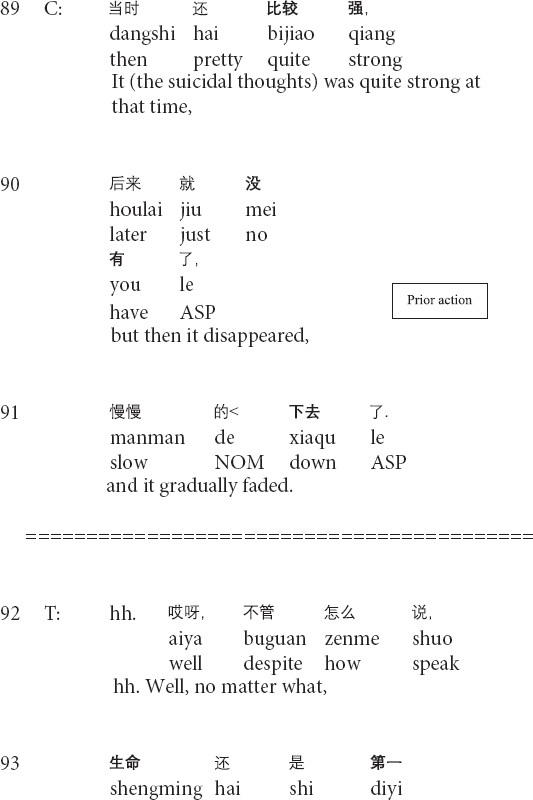



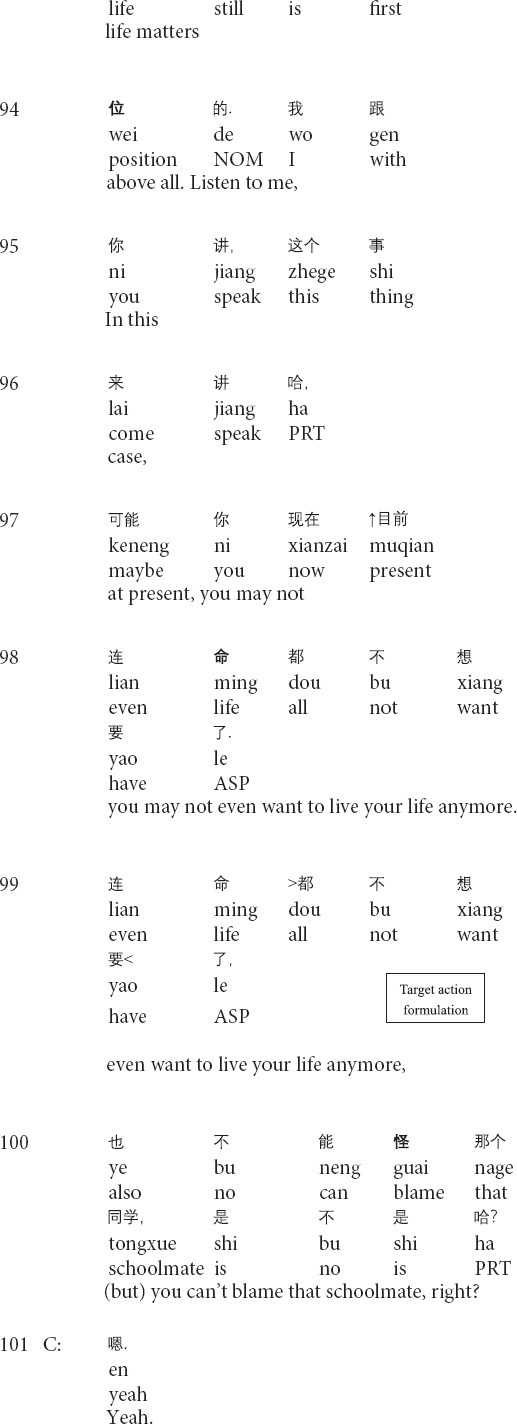



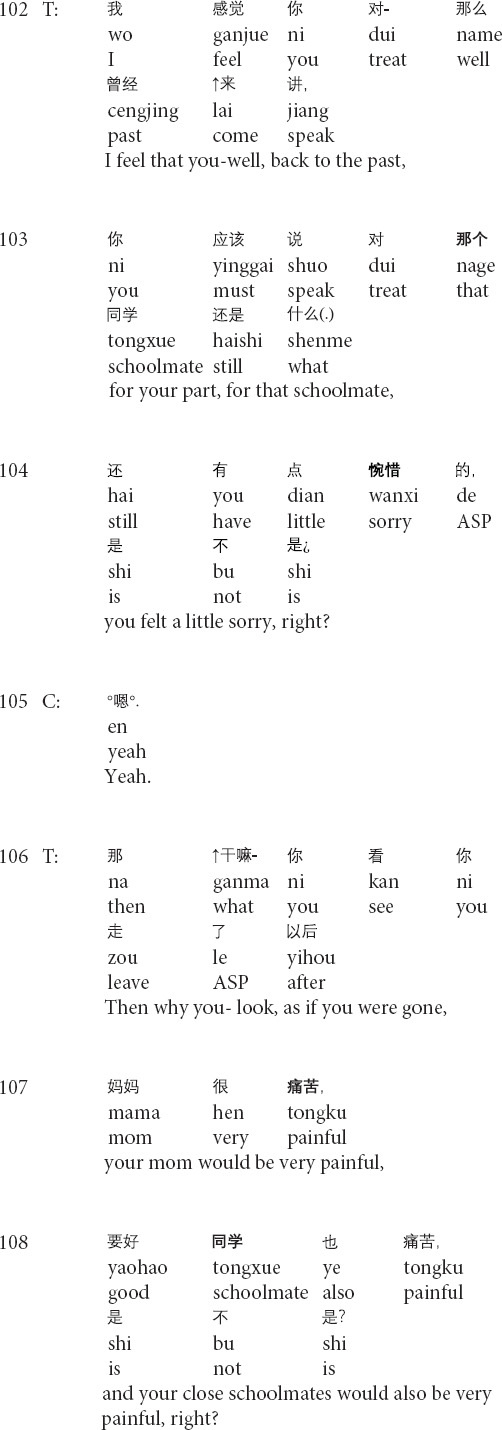



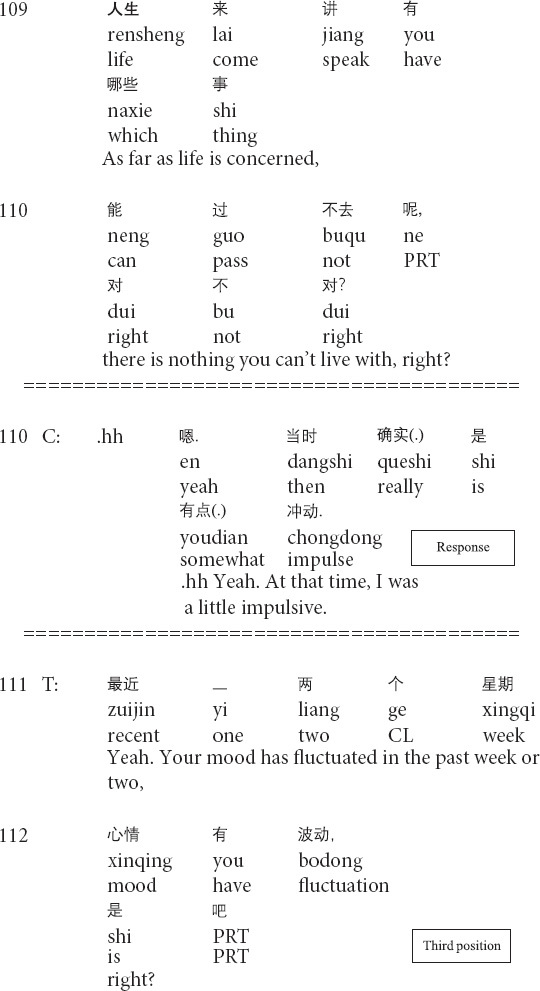


In the above Extract 3, the therapist’s formulation involves “a referential chain”: the focus of the objects that are referred to changes throughout the interaction (Peräkylä, 2019, p. 269). At the beginning of the formulation, the therapist focuses on the client’s thoughts about suicide (lines 95–98). Then, the therapist repeats his prior turn and shifts the focus of the referent from the client’s inner thoughts to the person in outer reality (i.e., his schoolmate) in lines 99–100. Though linking the client’s suicidal thoughts with the death of the client’s schoolmate, whose death leads to the client’s depression (not shown in this extract), the therapist interprets that the client should not attribute his suicidal thoughts to his schoolmate’s incident and solicits the client’s agreement by using the question tag *right?* (lines 99–100). After receiving the client’s confirming response, the therapist produces the second formulation of the client’s feelings about his schoolmate (i.e., feeling sorrow and sorry for his schoolmate, delivered before this extract) and shifts the referential focus to the client’s inner feelings. By invoking the client’s feelings about his schoolmate’s death, the therapist paves the way for depicting the future speculative scenario where the client commits suicide (line 106) and presenting the sorrow of the client’s mother and close schoolmates in the hypothetical scene (lines 107–108). In his third-position action (lines 111–112), the therapist does not provide any response to the client’s response. Instead, he starts to inquire about the client’s mood for the past two weeks. Therefore, the therapist transforms the referents in order to foreground particular aspects of the client’s narrative and gradually leads the client from noticing the outer behavior to facing his inner thoughts.

### Transformation of Emotion

As an essential aspect of psychotherapeutic interaction, the transformation of emotion has its therapeutic import in (helping clients) regulating and managing clients’ emotions, feelings, and moods. The shifts in emotional stance can occur in and through sequentially organized social actions, such as formulations, repair, and interpretations (Ekberg et al., 2016; Peräkylä, 2019). By mobilizing different resources, therapists can intensify or weaken the emotional import of the clients’ description to manage and regulate clients’ negative feelings and moods. The following Extract 4 demonstrates how the therapist weakens the client’s emotional expression. Before Extract 4, the client indicates her discomfort when talking to the present (male) therapist.

**Extract 4 (F17241: better mood)**


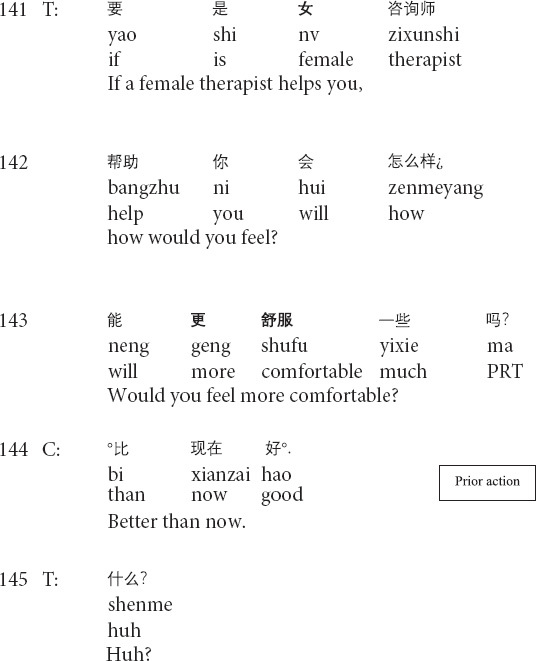



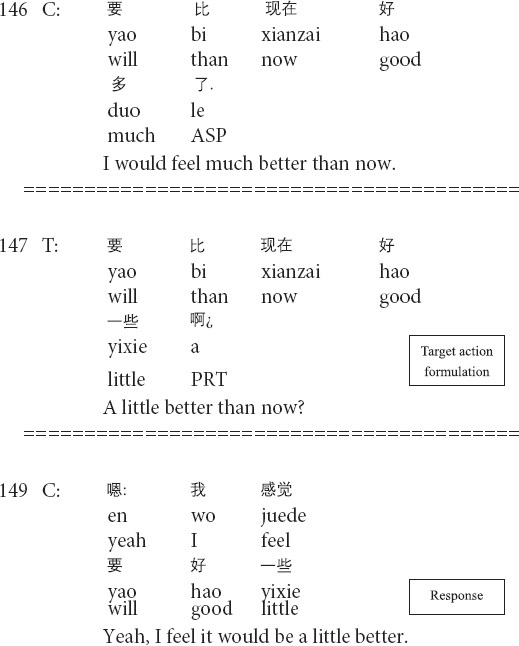


In lines 141–143, the therapist proposes a hypothetical scenario where the client talks with a female therapist and invites her to talk about her feelings. The prior action is concerned with the client’s emotional description of more comfort and better moods in case of talking with a female therapist. The target action in line 147 involves the therapist’s formulation as a therapist-initiated repair (Schegloff et al., 1977) by replacing the lexical terms *much* in line 146 with *a little* in line 147 and weakens the client’s emotional import. This turn is designed as an interrogative for downgrading the certainty and inviting the client’s agreement/disagreement. Then, in line 149, the client confirms the therapist’s formulation by providing the minimal acknowledgment token *yeah* and repeating the therapist’s words with mitigations by adding the marker *I feel*. The transformation of emotion is therapeutically meaningful in that the therapist downgrades the influence of outer reasons for the client’s discomfort by weakening her emotional description of talking with a female therapist and indicates the exploration of the inner causes for the client’s negative feelings and moods.

Similarly, in the following Extract 5, the prior action involves the client’s talking about her negative emotions and lousy mood when thinking about previous experiences (lines 66–69).

**Extract 5 (F17314: bad mood)**


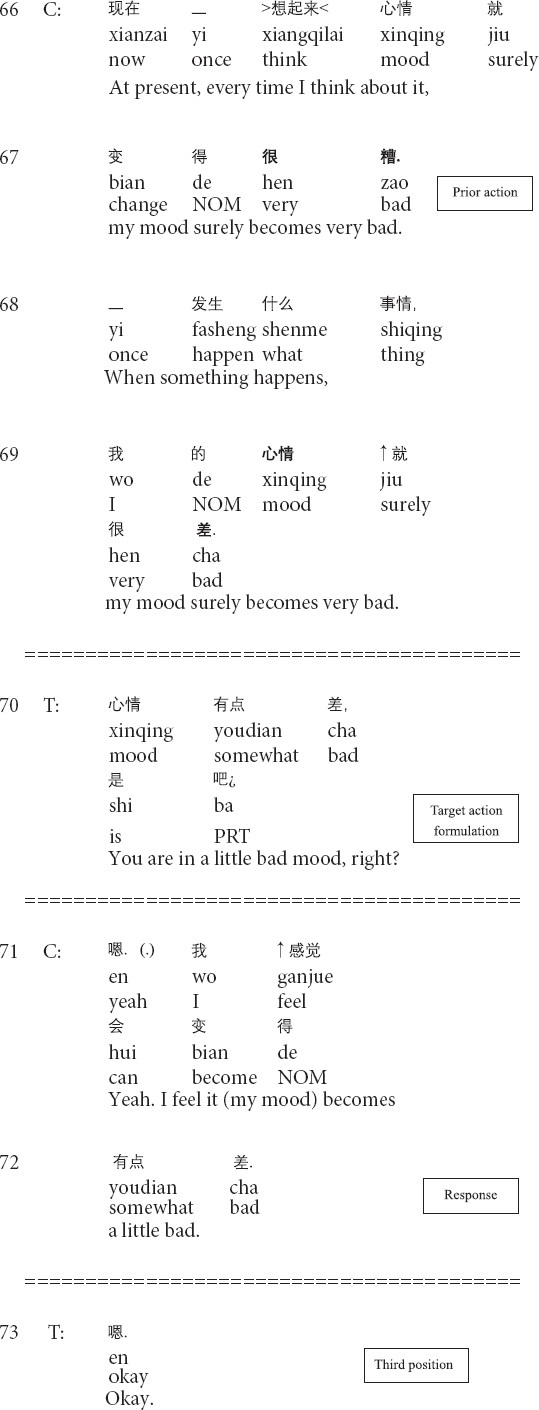


In line 70, the therapist produces the formulation by repeating the client’s prior words while weakening the description of the emotion (i.e., from *very bad* in line 69 to a little *bad* in line 70). By highlighting the client’s emotional feelings (see *highlighting formulation* in Weiste and Peräkylä, 2013), the therapist suggests that he recognizes the client’s negative emotions and the difficulties in handling her painful experiences and shows agreement. Further, by downgrading the emotional import of the client’s description, the therapist indicates that the client’s negative mood and painful experiences are less severe than what she previously described. The turn-final question tag, together with the formulation, invites the client’s agreement. In response, the client agrees with the therapist’s downgraded description by providing the minimal acknowledgment token *yeah* and introducing her judgment of her sensations with *I feel*, rather than simply repeating the therapist’s points of view (lines 72–73). In the third position, the therapist provides a minimal acknowledgment token to display the receipt of the client’s response. Therefore, the therapist’s transformation of emotion is therapeutically critical for showing the therapist’s understandings and agreement while presenting the client’s issue as less severe than the client believed.

### Transformation of Relation

The social relation between therapists and clients can be transformed moment by moment in the process of therapy sessions (Muntigl et al., 2013; Muntigl and Horvath, 2014b; Peräkylä, 2019). Therapists and clients collaboratively manage and transform their momentary relations, including (dis)agreement, and (dis)affiliation, through social actions, such as questions, formulations, and interpretations, in organized and systematic ways (e.g., MacMartin, 2008; Voutilainen et al., 2010; Ekberg and LeCouteur, 2015; Ekberg et al., 2016; Peräkylä, 2019). The following Extract 6 is an example of the transformation of relation, in which the therapist manages the client’s disaffiliation to his formulation by producing another formulation of the client’s disaffiliative response. Before this extract, the client describes that she feels unconformable and lacks the strength to move her body.

**Extract 6 (F21628: uncomfortable)**


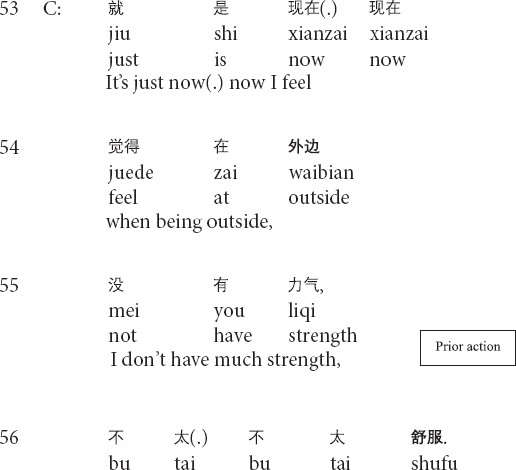



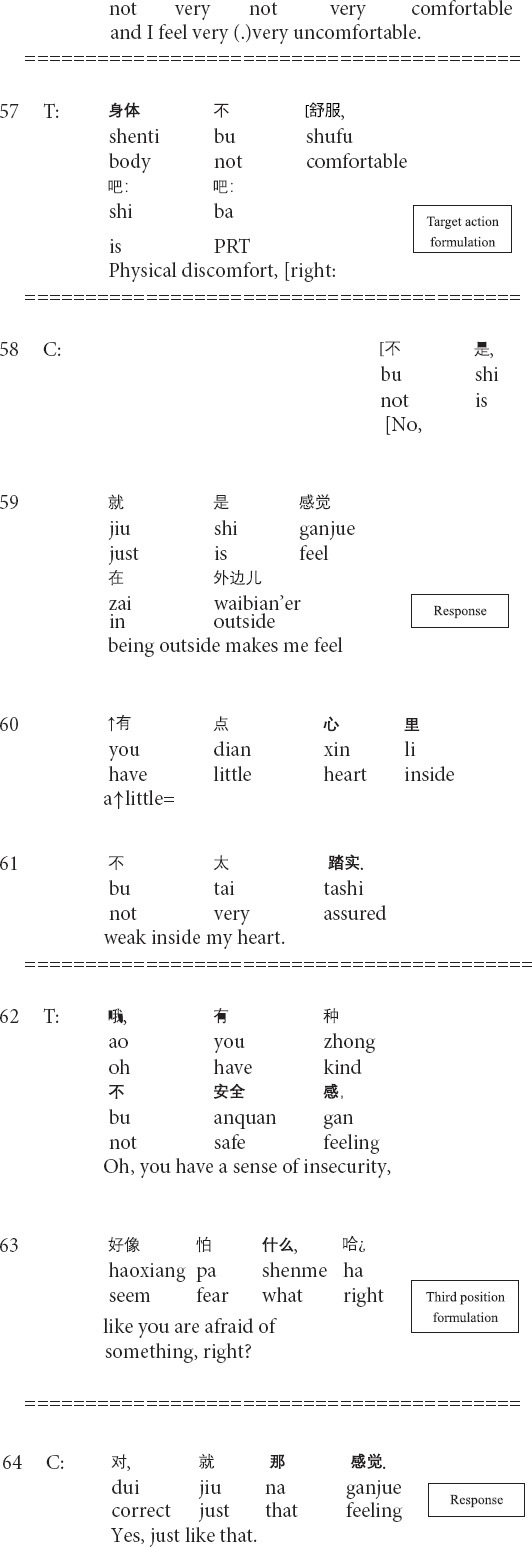


The therapist’s formulation in line 57 is characterized as a rephrasing formulation: the therapist presents his description of the client’s narrative in his own words (Bercelli et al., 2008; Weiste and Peräkylä, 2013). The therapist focuses on the client’s physical conditions and invites her to agree with a question tag *right*. However, the client does not confirm the therapist’s formulation. Instead, she negates it directly (line 58) and shifts the focus from physical weakness to psychological weakness. Then in the third position, the therapist produces another formulation of the client’s subjective experience just described by using the generic and abstract psychological expressions (line 62) and providing an explanation in layman’s terms (line 63). Furthermore, the final question tag *right?* clearly shows the therapist’s solicitation of the client to (dis)agreement. Through these designs of turns, the therapist manages the client’s disaffiliative response to his previous formulation and obtains the client’s re-affiliation (line 64). Through shifting back to affiliation, the therapist transforms the relation, which is therapeutically meaningful, as a means of achieving alignment and maintaining solidarity with the client and inviting the client to focus on painful experiences and negative moods.

## Discussion and Conclusion

The adjacent utterances (Schegloff, 2007) display the understandings of participants’ actions and embody clients’ experience, thus working as a vehicle for facilitating the transformation of clients’ experience (Peräkylä et al., 2008; Voutilainen et al., 2011; Bercelli et al., 2013; Peräkylä, 2019). Corroborated with the previous research on transformative sequences in psychotherapy (e.g., Voutilainen et al., 2011; Bercelli et al., 2013; Peräkylä, 2019), this study investigated the sequence organization of the psychotherapeutic processes and identified transformative sequences in the psychotherapeutic interaction with Chinese adolescents with depression.

By analyzing the transformative sequences in the psychotherapeutic interaction with Chinese adolescents with depression, our findings echoed and corroborated the work of Peräkylä (2019). However, unlike the model of Peräkylä (2019), we identified a new aspect of clients’ experience transformed by therapists: cognition. We further observed that in the third position, therapists could start new topics, such as inquiry about the current moods in Extract 3, without producing any response to the clients’ response.

Moreover, this study extended the understandings of the transformative sequences and demonstrated that the momentary changes in cognition, referents, emotions, and relations are involved and accomplished in and through the transformative sequences, thus helping clients face and overcome their emotional, cognitive, and psychological difficulties and problems. Furthermore, by transforming cognition, referent, emotion, and relation, therapists recognize clients’ issues and painful experiences and show agreement and empathy, thus maintaining solidarity with clients and steering the focus to clients’ subjective experiences.

The transformation of cognition involves the changes in the clients’ perceptions and understandings of their emotional, psychological and cognitive difficulties and problems, thus transforming their cognitive positions, particularly from being unknowing and unaware to being knowing and aware of their emotional and psychological issues (Extract 1). The transformation of cognition is therapeutically crucial for clients to know and face their psychological issues, thus paving the way for later management and treatment.

The referential nature of talk is particularly prominent in psychotherapeutic interaction (Peräkylä, 2011, 2019). Therapists and clients can use different referents when referring to particular aspects and objects of clients’ experiences. Therapists may shift the focus of attention by transforming referential terms moment by moment in and through sequentially organized actions during the psychotherapeutic process (Peräkylä et al., 2008; Bercelli et al., 2013; Voutilainen et al., 2018; Peräkylä, 2019). Therapists may adopt different methods to transform the referents in clients’ narratives, such as formulations and interpretations in Extracts 2 and 3 (see also Peräkylä, 2005, 2011). The referential transformation is psychotherapeutically meaningful as a means of showing therapists’ recognition and understandings of clients’ painful experiences and negative feelings and steering the focus to particular aspects of clients’ narratives.

The interactional work with clients’ emotions is a salient aspect of psychotherapeutic interaction (Peräkylä and Sorjonen, 2012; Stevanovic and Peräkylä, 2014; Peräkylä, 2019). Therapists regulate and transform clients’ emotions moment by moment in systematic and organized ways by mobilizing resources like prosody (Fitzgerald, 2013; Weiste and Peräkylä, 2013), crying (Muntigl and Horvath, 2014a), formulations (Extracts 4 and 5; see also *lexical substitutions* in Rae, 2008). The transformation of emotion is therapeutically meaningful in that it shows therapists’ understandings of clients’ painful experiences, and agreement with the emotions just described on the one hand. On the other hand, by downgrading clients’ emotional descriptions, therapists regard client’s problems as less severe than those described by clients (Extract 5), and indicate the exploration of the inner causes, rather than the outer reasons, for client’s negative feelings and moods (Extract 4), thus helping clients face their psychological difficulties and problems squarely.

The transformation of relations between therapists and clients takes place in moment-by-moment interaction (Ekberg et al., 2016; Peräkylä, 2019). Therapists and clients collaboratively manage and transform their social and emotional relations (e.g., (dis)agreement and (dis)affiliation) in the psychotherapeutic processes (e.g., MacMartin, 2008; Voutilainen et al., 2010; Muntigl et al., 2013; Muntigl and Horvath, 2014b; Ekberg and LeCouteur, 2015; Ekberg et al., 2016; Weiste et al., 2016). Therapists may adopt formulations to transform clients’ disaffiliation and retain affiliation in Extract 6 (see also Muntigl et al., 2013; Muntigl and Horvath, 2014b). The transformation of relations is therapeutically meaningful as a means of maintaining solidarity with the client and inviting the client to face and manage painful experiences and negative moods.

This study is not without limitations. First, the data set in our study is relatively small. Further studies are needed to collect more data from various hospitals in different locations. Second, since our data were audio-recorded, future studies could collect and analyze video-recorded data to explore the verbal and non-verbal resources mobilized by therapists and clients. Third, our study is the first to examine the transformative sequences in the psychotherapeutic interaction with Chinese adolescents with depression. More research is needed to investigate the structural and sequential organization of the therapist–client interaction (e.g., turn-taking, actions, and participation) in the psychotherapeutic process and examine the communication practices that therapists use to manage and transform clients’ experience.

## Data Availability Statement

The datasets presented in this article are not readily available because the data for the study consists of a corpus of audio-recorded psychotherapeutic interactions and is not allowed by the authors’ study protocol to share beyond the research team in order to protect the participants’ confidentiality. Questions regarding the datasets should be directed to WM, mawen@sdu.edu.cn.

## Author Contributions

WM contributed to conception and design of the study. XF collected the data. SZ and WM wrote the draft of the manuscript and made the most revisions. All authors contributed to manuscript revision, read, and approved the submitted version.

## Conflict of Interest

The authors declare that the research was conducted in the absence of any commercial or financial relationships that could be construed as a potential conflict of interest.

## Publisher’s Note

All claims expressed in this article are solely those of the authors and do not necessarily represent those of their affiliated organizations, or those of the publisher, the editors and the reviewers. Any product that may be evaluated in this article, or claim that may be made by its manufacturer, is not guaranteed or endorsed by the publisher.

## Footnotes

1
https://www.eva.mpg.de/lingua/resources/glossing-rules.php

## Appendix

### Appendix A

**The transcription conventions**

The transcription conventions used in this article follow those developed by Jefferson (2004), with some modifications.
[    The starting point of overlapped talks.
(.)  Just noticeable pause.
(0.5) Silence represented in tenths of a second.
::    The prolongation or stretching of the sound.
° °    Markedly softer talks.

**bold** Stress or emphasis.

>    The onset of markedly rushed or compressed talk.
<    The onset of markedly slowed or drawn out talk.
=    No interval between adjacent utterances by different speakers.
↑    Rising shifts in intonation.
¿    Intonations which rises more than a slight rise (,) but is not as sharp a rise as for a question mark.
